# Intelligent Discrimination of Grain Aging Using Volatile Organic Compound Fingerprints and Machine Learning: A Comprehensive Review

**DOI:** 10.3390/foods15020216

**Published:** 2026-01-08

**Authors:** Liuping Zhang, Jingtao Zhou, Guoping Qian, Shuyi Liu, Mohammed Obadi, Tianyue Xu, Bin Xu

**Affiliations:** 1 School of Food Science and Technology, Jiangnan University, Wuxi 214122, China; 13862450007@163.com; 2 Sinograin Zhenjiang Quality Inspection and Supervision Co., Ltd., Zhenjiang 212006, China; 15189185800@163.com (G.Q.); 18012327160@163.com (T.X.); 3 School of Food and Biological Engineering, Jiangsu University, Zhenjiang 212013, China; 19732578957@163.com (J.Z.); 1000006610@ujs.edu.cn (S.L.); obadialariki@gmail.com (M.O.)

**Keywords:** biomarkers, volatilomics, chemometrics, gas chromatography-mass spectrometry, grain storage stability

## Abstract

Grain aging during storage leads to quality deterioration and significant economic losses. Traditional analytical approaches are often labor-intensive, slow, and inadequate for modern intelligent grain storage management. This review summarizes recent advances in the intelligent discrimination of grain aging using volatile organic compound (VOC) fingerprints combined with machine learning (ML) techniques. It first outlines the biochemical mechanisms underlying grain aging and identifies VOCs as early and sensitive biomarkers for timely determination. The review then examines VOC determination methodologies, with a focus on headspace solid-phase microextraction coupled with gas chromatography-mass spectrometry (HS-SPME-GC-MS), for constructing volatile fingerprinting profiles, and discusses related method standardization. A central theme is the application of ML algorithms, including Partial Least Squares Discriminant Analysis (PLS-DA), Support Vector Machines (SVM), Random Forest (RF), and Convolutional Neural Networks (CNN)) for feature extraction and pattern recognition in high-dimensional datasets, enabling effective discrimination of aging stages, spoilage types, and grain varieties. Despite these advances, key challenges remain, such as limited model generalizability, the lack of large-scale multi-source databases, and insufficient validation under real storage conditions. Finally, future directions are proposed that emphasize methodological standardization, algorithmic innovation, and system-level integration to support intelligent, non-destructive, real-time grain quality monitoring. This emerging framework provides a promising powerful pathway for enhancing global food security.

## 1. Introduction

Grain crops such as wheat, maize, and rice are the primary sources of calories and nutrients for billions of people worldwide. Ensuring their quality and safety during postharvest storage is therefore essential for global food security and efficient resource utilization. During storage, grains inevitably undergo aging due to endogenous biochemical metabolism and external factors, including temperature, humidity, oxygen availability, and microbial activity [1,2,3]. This aging process is commonly manifested by increased hardness and acidity, loss of flavor and color, and reduced germination capacity [4]. These changes degrade organoleptic properties, nutritional value, and processing performance, and may be accompanied by mycotoxins accumulation, resulting in significant economic losses and food safety risks [5]. Consequently, accurate and efficient evaluation of grain freshness is critical throughout the grain supply chain. Current approaches for assessing grain aging rely primarily on traditional sensory evaluations and physicochemical measurements, such as fatty acid value, peroxide value, and germination rate [6]. Although widely used, these methods suffer from important limitations. They are often destructive and labor-intensive and, more importantly, act as lagging indicators that detect deterioration only after substantial quality loss has occurred, thereby limiting early warning capability. In addition, their dependence on subjective judgment and time-consuming procedures restricts their applicability for real-time, online, and intelligent monitoring in modern smart storage systems [6]. To overcome these limitations, increasing attention has been directed toward VOCs emitted during grain storage. The VOC profile, or volatilome, reflects dynamic biochemical processes associated with grain aging, including lipid oxidation, the Maillard reactions, and microbial metabolism [7,8]. Numerous studies have identified specific VOCs, particularly aldehydes and ketones such as hexanal, along with alcohols and esters, as sensitive biomarkers of aging progression. Notably, alterations in VOCs composition often occur before measurable changes in conventional quality indices, enabling early and non-destructive assessment of grain freshness [9]. Recent advances in hyphenated analytical techniques, particularly GC-MS, have facilitated high-throughput and high-resolution acquisition of VOC fingerprints [8]. However, these techniques generate high-dimensional and complex datasets, posing challenges for effective information extraction and model development. Early investigations applied multivariate statistical methods such as Principal Component Analysis (PCA) and PLS-DA, to achieve basic sample discrimination [10]. More recently, ML has emerged as a powerful alternative. ML algorithms are capable of capturing complex non-linear relationships within VOC datasets, automatically identifying informative features, and constructing robust classification models. As a result, they offer improved accuracy, automation, and reliability in grain aging assessment [10]. This review systematically summarizes recent progress in integrating GC-MS-based VOC fingerprinting with ML for intelligent discrimination of grain aging. Key methodological challenges are critically discussed, and future research priorities are proposed. The objective is to provide both a theoretical foundation and a practical roadmap for the development of next-generation intelligent grain quality monitoring systems.

## 2. Biochemical Mechanisms of Grain Aging and VOC Generation

### 2.1. Key Drivers of Grain Aging

#### 2.1.1. Endogenous Metabolism and Enzymatic Reactions

**Figure 1:**
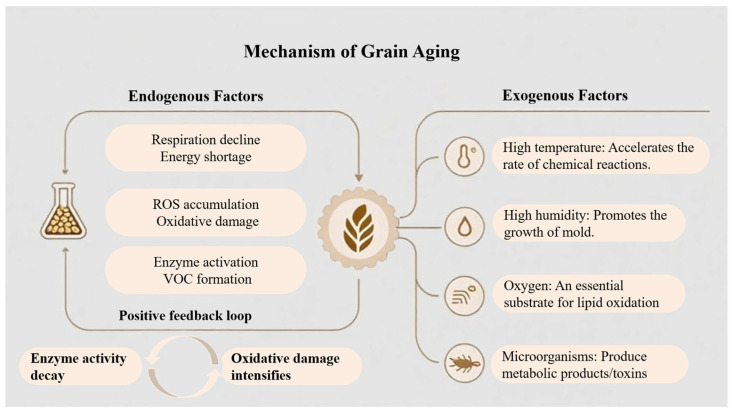
Schematic diagram of the mechanisms underlying grain aging.

Endogenous grain aging results from a complex interplay among declining respiratory, accumulation of oxidative stress, and progressive enzymatic degradation, as schematically illustrated in Figure 1.

Freshly harvested grains retain metabolic activity through respiration. Although respiration rates may temporarily increase under unfavorable conditions such as high moisture, insect infestation, or microbial proliferation, safe storage conditions generally lead to a gradual decline in endogenous respiration and overall metabolic intensity. This decline is associated with reduced activity of key enzymes involved in energy metabolism, including those in glycolysis and the tricarboxylic acid cycle. As a result, adenosine triphosphate synthesis capacity decreases, impairing nutrient utilization and increasing the vulnerability of mitochondrial membranes to damage. Mitochondrial dysfunction subsequently promotes continuous accumulation of reactive oxygen species (ROS), leading to sustained oxidative stress [11], as shown in Figure 1. Under these conditions, endogenous antioxidant defense systems, including superoxide dismutase (SOD), catalase (CAT), and glutathione peroxidase (GSH-Px), become insufficient to neutralize excessive ROS [12]. The resulting free radicals induce oxidative damage to membrane lipids, proteins, and nucleic acids. Among these processes, lipid peroxidation plays a particularly critical role because it directly compromises cellular integrity while generating substrates for downstream reactions. Lipoxygenase (LOX) is widely recognized as a key enzyme in grain aging. It catalyzes the oxidation of unsaturated fatty acids such as linoleic and linolenic acids, forming lipid hydroperoxides that decompose into characteristic VOCs, including hexanal, heptanal, and nonanal. These compounds are major contributors to stale and off-flavors in aged grains. During prolonged storage, the concentration of free fatty acids typically increases, providing additional substrate for LOX activity and further accelerating the accumulation of undesirable volatiles [13]. In parallel, elevated activities of polyphenol oxidase (PPO) and peroxidase (POD) promote the oxidation of polyphenolic compounds into quinones, which subsequently polymerize to form brown pigments and cause grain darkening [12]. Other enzymatic pathways also contribute to quality degradation. Amylase activity may be transiently enhanced during early storage, partially hydrolyzing starch into dextrins and reducing sugars. These products alter texture properties and act as precursors for Maillard reactions, as well as substrates for microbial metabolism. As aging progresses, overall enzyme activity declines and starch crystallinity decreases, resulting in harder and less elastic texture after cooking [14]. Protease activity often increases during the early stages of storage, promoting the gradual hydrolysis of storage proteins into peptides and free amino acids. Some nitrogen-containing degradation products are further converted into volatile amines and alcohols, intensifying bitter and musty off-odors [15]. Collectively, these biochemical processes form a reinforcing cycle in which substrate depletion, enzymatic dysfunction, and oxidative damage mutually intensify. This progressive destabilization of metabolic homeostasis ultimately leads to pronounced deterioration of grain quality and flavor.

#### 2.1.2. Exogenous Environmental Stress and the Impact of Microorganisms and Pests

External abiotic factors, including temperature, humidity, light, and oxygen availability, exert strong control over the quality and stability of stored grains. Elevated temperature accelerates lipid oxidation and alters enzymatic activity, thereby increasing the rates of key biochemical reactions associated with aging [16]. For example, in high-quality indica rice, each 5 °C increase in storage temperature significantly increased fatty acid value and malondialdehyde content, while sensory scores and cooked rice hardness declined [17]. Similarly, maize stored at 30 °C for 12 months exhibited an approximately 40% reduction in ethanol fermentation efficiency, indicating that prolonged exposure to high temperature amplifies metabolic deterioration [18]. Relative humidity (RH) is another critical determinant of grain stability. Elevated moisture facilitates water redistribution and starch retrogradation and promotes fungal growth, leading to the accumulation of fungal metabolites and mycotoxins [19]. When RH increased from 55% to 75%, the germination rate of maize decreased from 90% to 60%, while *Aspergillus flavus* infection increased fourfold [20]. Oxygen availability further contributes to deterioration by serving as a substrate for lipid auto-oxidation. Under aerobic conditions, free-radical chain reactions continuously generate ROS as well as volatile aldehydes and ketones that degrade grain quality [21]. Light exposure represents an additional degradation pathway through photo-oxidation. Light promotes free radical formation in unsaturated fatty acids, initiating self-propagating oxidation reactions. These processes generate hydroperoxides and secondary products such as aldehydes and ketones, which subsequently promote protein oxidation and aggregation. As a result, grain flavor, color, and nutritional value are progressively compromised [22]. Biotic factors further intensify these effects through synergistic interactions. Insect infestation causes mechanical damage and releases metabolic heat, creating favorable conditions for fungal colonization. In turn, fungi secrete hydrolytic and oxidative enzymes that accelerate lipid oxidation and protein degradation. This positive feedback between insect activity and microbial metabolism markedly hastens the transition from fresh to deteriorated grain states [23,24]. In summary, grain aging is a multifactorial process driven by the combined effects of temperature, humidity, oxygen, light, and biological agents. External stressors initiate and accelerate endogenous oxidative reactions, while biological activity amplifies chemical degradation through physical damage and enzymatic catalysis. The convergence of these factors results in systemic deterioration of grain structure and composition, manifested as lipid peroxidation, protein denaturation, and loss of flavor, and reduced storage stability and nutritional quality.

### 2.2. Traditional Methods for Assessing Grain Aging and Their Limitations

In practice, grain aging has traditionally been evaluated using a combination of physicochemical and organoleptic methods. Organoleptic assessment relies on manual observation of changes in color, odor, and texture. For example, aged rice often develops a yellowish-brown appearance and a musty odor, while flour produced from aged wheat appears duller and emits a stale smell. Duizer [25] discussed the application of sensory science in evaluating grain-based foods. However, despite their simplicity and intuitive nature, sensory methods are inherently subjective and lack the precision required for quantitative assessment. Physicochemical analyses provide more objective indicators of aging progression. The acidity indicator method uses pH-sensitive dyes to distinguish rice freshness, with fresh grains producing a green coloration and aged grains appearing orange-red [26]. Nevertheless, endpoint determination remains visually based and is therefore prone to operator bias. The electrical conductivity method evaluates cell membrane integrity by measuring the conductivity of a grain steep solution. Increased conductivity in aged maize reflects enhanced ionic leakage, although the results can be influenced by temperature variation and water quality [27]. Additional conventional techniques include monitoring respiration intensity through CO_2_ release to assess metabolic decline and measuring fatty acid value to quantify free fatty acid accumulation associated with lipid hydrolysis and oxidation. While these indicators are useful for quality control, they capture only limited aspects of the aging process. Several inherent limitations restrict the effectiveness of these traditional approaches. First, most rely on endpoint indicators that become detectable only after substantial physicochemical deterioration has occurred, limiting their sensitivity to early-stage aging [28]. Second, conventional physicochemical markers typically target a single class of metabolites and therefore fail to represent the broader molecular changes that develop during storage [29]. Third, many methods are labor-intensive, time-consuming, and dependent on skilled operators, which reduces their suitability for rapid, high-throughput, and real-time monitoring applications [30]. To overcome these constraints, increasing research attention has shifted toward VOCs released during grain storage. VOCs provide a dynamic reflection of underlying molecular transformations and, due to their specificity and quantifiability, offer strong potential as biomarkers for early, rapid, and intelligent monitoring of grain aging.

### 2.3. Formation Pathways of VOCs and Their Potential as Indicators of Grain Aging

**Figure 2:**
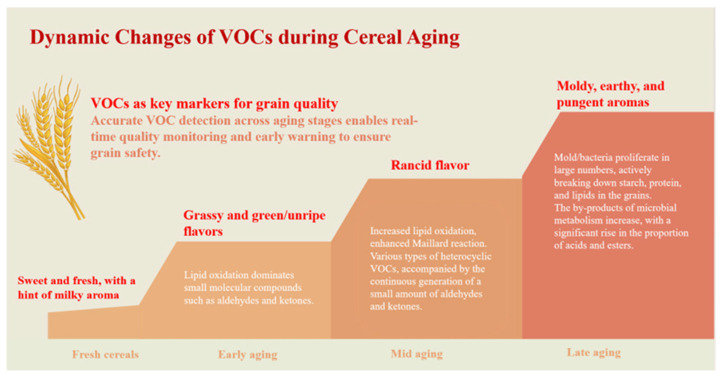
Dynamic changes in VOCs during grain aging.

Across different stages of grain aging, the generation of VOCs follows a characteristic dynamic pattern, as illustrated in Figure 2. During the early stages, lipid oxidation predominates. Unsaturated fatty acids undergo enzymatic or non-enzymatic oxidative cleavage, producing low-molecular-weight volatiles such as aldehydes and ketones [31]. Among these compounds, hexanal is widely recognized as a key biomarker of early aging due to its rapid accumulation and early appearance during storage [32]. As storage progresses, and under favorable temperature and moisture conditions, the Maillard reaction becomes increasingly active. Reducing sugars react with amino acids to form a range of heterocyclic compounds, including pyrazines, furans, and pyrroles, as well as additional aldehydes. These shifts in dominant reaction pathways drive a transition in aroma characteristics from fresh and grassy to roasted, nutty, and ultimately stale notes [33,34]. Poorly controlled storage conditions, particularly elevated temperature and humidity, further promote microbial growth. Molds and bacteria contribute to grain deterioration by degrading starch, proteins, and lipids, leading to the formation of alcohols, organic acids, and esters [35]. Together with products of lipid oxidation and Maillard reactions, these microbial metabolites accelerate the development of rancid and musty off-flavors [9]. Because VOCs directly reflect the underlying biochemical and microbial processes associated with aging, they serve as sensitive and specific biomarkers for grain quality monitoring. Changes in VOCs composition often occur before detectable variation in conventional physicochemical indices, enabling earlier assessment of deterioration [36]. This sensitivity arises from the fact that VOCs act as direct proxies for defined reaction pathways. For instance, hexanal, a primary product of lipid oxidation, typically increases earlier and more markedly than traditional quality indicators. In grains rich in polyunsaturated fatty acids, such as oats, this increase frequently precedes measurable changes in standard freshness metrics [37]. Moreover, VOC profiles can capture concurrent deterioration mechanisms that are not resolved by single physicochemical parameters. Under high-temperature and high-humidity conditions, lipid oxidation and microbial metabolism may proceed simultaneously. The resulting VOC spectrum therefore includes not only aldehydes and ketones but also characteristic microbial metabolites, such as 3-methyl-1-butanol and phenylethyl alcohol [38]. Analysis of the relative abundance and temporal evolution of these VOC classes enables discrimination between oxidation-driven aging and microbial spoilage, a distinction that cannot be achieved by measurements such as acid or peroxide values alone.

Compared with conventional physicochemical indicators, including moisture content, free fatty acid value, and germination rate, VOC profiling is capable of detecting early biochemical and microbial changes that precede pronounced quality loss. Minor shifts in VOC composition should therefore be interpreted as early-warning signals rather than indicators of mild deterioration. By enabling earlier identification of storage-related risks, VOC monitoring can identify storage-related risks at earlier stages and shorter storage durations, supports timely intervention to prevent irreversible quality degradation, although the effective lead time depends on grain type, storage conditions, and analytical sensitivity.

### 2.4. Research Progress and Challenges in VOC-Based Grain Aging Discrimination

Recognizing of the potential of VOCs for discriminating grain aging has stimulated extensive research worldwide, leading to substantial progress in biomarker identification, differentiation of storage states, and simulation of deterioration processes. Numerous studies have demonstrated that specific VOCs serve as reliable proxies for grain aging and spoilage. Hexanal, a canonical product of lipid oxidation, has been consistently validated as a dominant early-stage marker in maize, rice, and wheat [39,40,41]. In addition, compounds such as 1-octen-3-ol and 3-methyl-1-butanol, often referred to as mushroom alcohols, are frequently associated with fungal activity and accumulate rapidly during the onset of microbial spoilage. These compounds therefore function as effective early-warning indicators of biological deterioration [42]. Collectively, these findings support the development of rapid analytical strategies that focus on a limited set of representative VOC biomarkers. For qualitative discrimination of storage states, HS-SPME combined with GC-MS or gas chromatography-ion mobility spectrometry (GC-IMS) is widely employed to acquire high-dimensional VOC fingerprints. These datasets are commonly analyzed using multivariate statistical methods, including PCA and PLS-DA [43]. This analytical framework enables reliable discrimination between fresh and aged grains and can also identify accelerated aging (AA) conditions induced by elevated temperature and humidity. In rice, VOC fingerprinting has additionally been applied to trace geographical origin across premium and common cultivars, demonstrating high sensitivity and resolution for characterizing both storage status and provenance [44]. Simulation and monitoring of specific deterioration pathways are predominantly conducted under controlled laboratory conditions. By independently or jointly manipulating environmental variables such as temperature, humidity, oxygen concentration, and microbial inoculation, researchers have systematically characterized the dynamic evolution of VOC profiles during storage [21,45,46]. For example, elevated temperatures have been shown to markedly accelerate the formation of aldehydes and ketones associated with lipid oxidation in rice, highlighting the central role of temperature in driving aging-related VOC generation [47]. Together, these studies provide critical mechanistic insights into environment-dependent VOC release and form an important foundation for the development of predictive models aimed at intelligent grain storage management.

## 3. VOC Determination and Identification Technologies and the Challenge of Data Standardization

### 3.1. Analytical Methods for VOC Profiling

The analysis of VOCs in stored grains presents several technical challenges, including high chemical diversity, a wide concentration range, and the difficulty of detecting low-abundance compounds within complex matrices. As a result, analytical methods must provide high sensitivity, strong separation capability, and sufficient throughput. Among available approaches, chromatographic techniques are the most robust and widely adopted for generating comprehensive VOC fingerprints for freshness evaluation, owing to their superior resolution and analytical sensitivity [36]. GC and its hyphenated variants are currently the most extensively applied tools in grain VOC research. GC with flame ionization detection (GC-FID) offers high sensitivity and is well-suited for quantitative analysis; however, it does not provide structural information for compound identification [48]. Gas chromatography-olfactometry (GC-O) enables direct identification of odor-active compounds, but the method is inherently subjective and difficult to standardize across laboratories [49]. GC-IMS allows rapid screening and pattern recognition, although its application is often constrained by dependence on reference databases and relatively limited separation resolution [50]. In contrast, GC-MS integrates high-performance chromatographic separation with mass-based detection, enabling both accurate quantification of target compounds and structural elucidation of unknown volatiles through mass spectral analysis. Owing to its favorable balance of sensitivity, selectivity, and versatility, GC-MS has become the cornerstone analytical platform for VOC profiling in studies of grain aging and freshness evaluation [51,52]. A comparison of these major analytical techniques is summarized in Table 1.

### 3.2. HS-SPME for Sample Preparation

Efficient extraction and accurate analysis of VOC biomarkers are essential for constructing reliable chemical fingerprints of grain aging. HS-SPME, most commonly coupled with GC-MS, is a key technique in this analytical workflow. As a solvent-free sample preparation method, HS-SPME integrates enrichment and transfer of target analytes from complex matrices into a single step, thereby reducing matrix interference and improving analytical throughput. Compared with conventional approaches such as liquid–liquid extraction or solvent-assisted extraction, HS-SPME offers greater operational simplicity, high sensitivity, and good reproducibility, which has led to its widespread application in food flavor and safety research [56]. In grain studies, HS-SPME–GC-MS has been extensively applied for comprehensive VOCs profiling during aging. For example, optimization of HS-SPME conditions has enabled quantitative determination of 2-acetyl-1-pyrroline (2-AP) in rice, with detection limits in the ng/kg range [57]. This approach has also been used to monitor volatile changes across rice ripening stages. Notably, 2-AP is also a key aroma compound present in finished bread products. When combined with relative odor activity values (ROAV), it identified 65 key compounds; (Z)-6-nonenal and (Z,Z)-3,6-nonadienal differentiated ripening stages, while 2-AP, heptanal, and nonanol distinguished aromatic from non-aromatic rice varieties [42]. These findings demonstrate the suitability of HS-SPME for capturing both early-stage lipid oxidation products and the subtle flavor compounds associated with later stages of aging. Several parameters critically influence HS-SPME extraction efficiency, including fiber coating type, extraction temperature, and extraction time. Different fiber coatings exhibit distinct selectivity. Carboxen/Polydimethylsiloxane (CAR/PDMS) is particularly effective for low-molecular-weight aldehydes and ketones, whereas Divinylbenzene/Carboxen/Polydimethylsiloxane (DVB/CAR/PDMS) provides broader coverage and improved performance for mid- to high-molecular-weight aldehydes, ketones, esters, and organic acids [58,59]. In addition, intrinsic properties of the grain matrix, such as moisture content, lipid composition, and starch structure, affect volatile release kinetics into the headspace and therefore influence optimal extraction conditions [60,61]. For grain aging studies, systematic optimization of HS-SPME parameters is therefore required for each grain type and target compound class. Such optimization is necessary to balance efficient capture of early aldehyde and ketone biomarkers with later-stage ester and acid markers associated with advanced deterioration.

### 3.3. The Core Role of GC-MS in Qualitative and Quantitative VOC Analysis

GC-MS is the central analytical platform for both qualitative identification and quantitative determination of volatile biomarkers. Its high-efficiency chromatographic separation combined with mass-based detection enables reliable resolution of diverse VOC classes, including aldehydes, ketones, alcohols, esters, and organic acids, even within complex grain matrices [54]. To enhance sensitivity for trace-level analytes, selected ion monitoring (SIM) mode is frequently applied. When combined with internal or external standards, SIM can achieve detection limits at the ng/g level [62]. This high-sensitivity strategy has been successfully used, for example, to detect aromatic adulterants in fragrant rice, with limits of detection as low as 0.5–10 ng/mL [55]. GC-MS has also been integrated with complementary sensing approaches. The combination of GC-MS and electronic nose (e-nose) technology has been employed to characterize VOC release patterns under different maize storage conditions, providing insight into optimal environmental parameters for quality preservation [63]. Reliable VOC analysis using GC-MS depends on careful standardization and optimization of analytical conditions. The grain matrix, such as maize, wheat, or rice strongly influences VOC release behavior and analytical performance. As a result, parameters including capillary column selection, carrier gas flow rate, and oven temperature programming must be tailored to the specific sample type to minimize analytical artifacts such as co-elution and signal masking. Matrix-dependent differences in lipid content illustrate this requirement. The relatively high lipid content of maize often necessitates higher equilibration temperatures, typically 60–70 °C, to promote efficient VOC re-lease [64]. In contrast, the lower fat content of wheat allows more rapid liberation of aldehydes and ketones, making lower temperatures in the range of 50–60 °C more suitable [65]. Despite its strong separating capability and reliable compound identification through mass spectral library matching, GC-MS has inherent limitations. Thermolabile or highly polar compounds, such as polyphenols, often require derivatization or complementary analytical techniques, including liquid chromatography (LC). Co-elution and matrix interference may still compromise quantitative accuracy, particularly in complex samples. In addition, GC-MS analysis typically requires several tens of minutes per run, limiting sample throughput. Both instrument operation and data interpretation demand substantial expertise and access to high-quality spectral libraries. Ongoing developments in comprehensive two-dimensional gas chromatography (GC × GC-MS), automated high-throughput platforms, and advanced data-mining algorithms are expected to further enhance the applicability and intelligence of GC-MS for grain aging assessment and quality control.

### 3.4. Technical Limitations and the Need for Standardization

Although the HS-SPME-GC-MS workflow provides a powerful approach for profiling VOCs during grain aging, its broader application is constrained by several technical limitations, particularly those related to methodological standardization. Sample preparation plays a decisive role in extraction efficiency and volatilome coverage, and HS-SPME performance is highly sensitive to parameters such as fiber coating, extraction temperature, extraction time, and agitation conditions [66,67]. As a result, method optimization is often matrix-specific and requires extensive empirical adjustment. Substantial inter-laboratory variability further limits comparability. Differences in chromatographic columns, carrier-gas conditions, and mass spectrometer configurations can lead to significant variation in VOC profiles, reducing reproducibility across platforms and studies [68]. At the data-processing stage, GC-MS datasets are characterized by complex peak structures and frequent co-elution, necessitating multi-step workflows for peak detection, deconvolution, and retention-time alignment [69,70]. Variability in spectral quality across instruments also complicates compound identification based on library matching [71,72]. Together, these methodological and analytical inconsistencies hinder robust comparison of VOC datasets and limit the transferability of results. These challenges underscore the urgent need for standardized analytical protocols and harmonized data-processing workflows to support reproducible, scalable, and reliable VOC-based grain aging assessment.

## 4. ML Strategies and Algorithm Selection for VOC Fingerprint-Based Modeling

### 4.1. The Multi-Dimensional Nature of VOC Data and Its Suitability for ML

VOC data acquired from GC-MS are inherently multi-dimensional and semi-structured. Typically, variables include retention times, peak areas, and mass spectral features associated with numerous [44]. These characteristics arise from the complex biochemical degradation pathways involved in grain aging, in which the simultaneous breakdown of lipids, carbohydrates, and proteins produces a diverse array of metabolites, as illustrated in Figure 1. Each sample can therefore be represented as a chemical fingerprint vector, with individual features corresponding to specific chemical substances. This direct link to identifiable compounds provides a high degree of interpretability and traceability [73]. As shown in Figure 2, grain aging introduces distinct, time-dependent patterns into VOC datasets. For example, lipid degradation and subsequent oxidation lead to the progressive accumulation of aldehydes and ketones [74]. These processes leave measurable molecular signatures in the VOC profile, enabling discrimination among samples at different aging stages [73]. However, VOC datasets are typically high-dimensional, non-linear, and partially redundant. These properties reduce the effectiveness of conventional linear analytical methods. ML is well suited to address these data characteristics. Unlike traditional methods that rely on linear assumptions or predefined thresholds for single analytes, ML uses a data-driven framework to model complex relationships between the full VOC feature set and grain aging status. This capability is supported by the universal approximation theorem, which states that neural networks can approximate continuous functions to an arbitrary level of accuracy when appropriate model architectures and optimization strategies are applied [75,76]. Accordingly, advanced ML structures, including cascade neural networks, have been used to map multi-dimensional VOC signals to quantitative quality indicators [77]. In practical applications, ML algorithms can automatically perform feature selection and dimensionality reduction, isolating informative biomarkers such as hexanal or 1-octen-3-ol from background noise. At the same time, they construct non-linear decision boundaries that enable accurate discrimination among aging stages or even between different deterioration mechanisms, such as oxidative versus microbial processes [78]. ML therefore provides a powerful framework for extracting deeper informational value from VOC fingerprints and forms an essential methodological basis for developing robust predictive models for grain aging assessment.

### 4.2. Performance Comparison and Selection of Mainstream ML Algorithms

VOC data derived from GC-MS are characterized by many variables, complex spectral profiles, and frequent peak overlap [79]. This complexity reflects the underlying biochemical networks shown in Figure 1, where multiple metabolic pathways (e.g., lipid oxidation, protein hydrolysis) operate simultaneously and generate highly correlated features. Traditional approaches based on manual interpretation or single-variable analysis are inefficient and subjective when applied to such high-throughput data. They also struggle to identify latent discriminatory patterns arising from interactions among variables [80]. To improve data-mining efficiency, ML methods are increasingly used to model non-linear relationships and to construct robust classifiers for grain aging status. Existing ML implementations can broadly be grouped into shallow learning and deep learning (DL). Because they require smaller sample size and offer greater interpretability, shallow learning algorithms remain the dominant tools in grain-aging research. The ML algorithms discussed in this review were selected based on their demonstrated suitability for typical VOC data characteristics, including high dimensionality, multicollinearity, non-linear structure, and relatively limited sample sizes, as well as their widespread use in VOC-based grain quality and aging studies [81]. Accordingly, this review focuses on five representative model families: exploratory linear methods such as PCA combined with Linear Discriminant Analysis (LDA); supervised classifiers such as PLS-DA and SVM; ensemble learning approaches including RF and XGBoost, as well as DL models [82]. A comparative summary of the main ML models applied in VOC-based discrimination is provided in Table 2.

PCA-LDA and PLS-DA are most effective when the research objective is biomarker screening and exploratory analysis in high-dimensional datasets by identifying directions of maximum inter-class variance [83,84,85]. These linear methods are particularly suitable when aging signals exhibit high contrast, allowing efficient feature compression. A key distinction is that PCA-LDA is best suited to datasets in which class boundaries are already well separated, whereas PLS-DA incorporates class information and can therefore detect inter-class differences more sensitively [94]. For instance, Lasalvia successfully applied PCA-LDA to complex FTIR spectra [95], while Foroozani reported that PLS-DA achieved significantly better performance in classifying wheat varieties [96]. The main limitation of both approaches is the linearity assumption, which can result in information loss when modeling the complex, non-linear degradation patterns typical of rice or wheat. SVM is particularly well-suited to high-dimensional, small-sample (HDLSS) problems and performs robustly when class boundaries are relatively clear [86,87]. This makes it a common choice for laboratory-scale studies, such as maize storage classification across different storage years [64]. Its key strength lies in resistance to overfitting under data-scarce conditions. However, SVM performance is highly sensitive to parameter tuning, including kernel selection and the cost parameter C, and computational complexity can increase rapidly with data size [86,87]. Ensemble learning approaches, specifically RF and XGBoost, are preferred when predictive stability and the ability to model non-linear features are priorities. RF provides embedded variable selection and is robust to noise [88,89]. This capability has enabled near error-free geographical traceability of rice based on key VOC biomarkers [43]. XGBoost extends this capability through iterative gradient-boosting optimization and strong performance with complex, interacting variables [90,91], and has been proposed for rice variety classification using “deeply typed” pipelines [97]. However, ensemble learning models are generally less interpretable, which can limit their usefulness for explaining biological mechanisms. Compared with shallow methods, DL models (e.g., 1D-CNNs) enable automated, end-to-end learning directly from raw spectral or chromatographic waveforms, avoiding bias introduced by manual feature extraction [92,93]. DL approaches have demonstrated high efficiency and sensitivity in THz absorption spectra [98] and GC-MS breath-analysis studies [99]. However, DL models are constrained by their limited interpretability, their large training-data requirements, and frequent convergence challenges when applied to sparse or zero-inflated GC-MS outputs [100]. As a result, although DL offers strong engineering potential, shallow ML methods continue to dominate VOC-based grain aging discrimination because they perform more reliably under limited-sample conditions while retaining interpretability.

### 4.3. Species-Specific VOC Profiles and Their Impact on Modeling Strategies

The mechanisms of VOC generation during grain aging are influenced by species-specific factors, including the anatomical distribution of lipids, enzyme activities, and characteristic aroma precursors. These chemical differences directly shape VOC data structures and therefore affect the choice of modeling strategies. Maize contains a relatively large embryo (germ), a which represents approximately 10–12% of the kernel weight and is enriched in unsaturated fatty acids. As a result, maize aging is often dominated by intensive lipid oxidation, leading to the pronounced accumulation of aldehydes and ketones such as nonanal, hexanal, and 2-heptanone [101,102]. Because these VOC signals are strong and consistent, they are readily captured by linear chemometric approaches. Consequently, high classification performance has frequently been reported using linear methods such as PCA followed by LDA, or PLS-DA [8]. In contrast, VOC changes during rice and wheat aging are generally more subtle and dependent on chemical composition, which requires more sensitive feature extraction strategies. For rice, particularly aromatic varieties, degradation of 2-AP—the compound responsible for the characteristic popcorn-like aroma—is a key indicator of quality loss. This decline is often accompanied by increasing concentrations of hexanal and pentanal [57,103]. However, reductions in desirable aroma compounds and the emergence of staling markers do not always follow linear trends. As a result, non-linear ML algorithms such as SVM or RF often provide better discrimination performance for VOC-based rice aging assessment. Wheat aging produces even weaker VOC signals, which are frequently dominated by trace-level compounds such as (E)-2-nonenal, a major contributor to cardboard-like off-flavor [104,105]. Due to signal overlap and low analyte concentration, these datasets can be challenging for shallow linear models. In larger datasets, advanced modeling strategies, including DL architectures, may therefore be advantageous for detecting latent VOC patterns that are difficult to resolve using conventional approaches [106,107,108]. A summary of these species-specific characteristics and modeling strategies is provided in Table 3.

### 4.4. Current Modeling Bottlenecks and Generalization Challenges

Despite the promising performance of ML in VOC-based discrimination of grain aging, several algorithmic and data-related limitations remain. Because natural aging is slow, most studies rely on AA experiments, resulting in datasets that are typically small and highly dimensional. In these high-dimension, low-sample-size (HDLSS) settings, conventional feature selection approaches may produce unstable results, and even apparently well-performing algorithms may fail to capture the true underlying structure of the data [109,110]. DL models are particularly susceptible to overfitting under such conditions due to their large parameter space [111]. GC-MS-derived VOC datasets also commonly contain many correlated or redundant variables, while only a limited subset is directly associated with aging status [112]. This redundancy increases computational burden and can introduce noise that degrades model generalization performance [113]. As a result, overall model accuracy depends strongly on the quality of feature extraction and preprocessing, highlighting the need for close coordination between analytical method development and data-analysis workflows [114,115]. Overall, these issues indicate that ML performance in VOC-based grain aging studies is constrained not only by algorithm selection but also by intrinsic data structure and feature quality. Prospective algorithmic innovations and strategies for bridging the gap between laboratory data and natural aging (NA) in real-world storage environments are discussed further in Section 5.2.

## 5. Research Challenges and Future Directions

### 5.1. Technical Bottlenecks: Standardization VOC Determination and Construction Multi-Source Database

For VOC fingerprinting to transition successfully from laboratory research to industrial application, the most critical challenge is the lack of standardized and comparable data. Unified standard operating procedures (SOPs) covering the entire analytical workflow, including sample preparation, instrumental analysis, and data preprocessing, are essential to minimize inter-laboratory variability and improve reproducibility. Without such standardization. VOC datasets generated by different research groups remain fragmented and difficult to integrate. In parallel, the development of large-scale, multi-source VOC databases is a prerequisite for robust modeling and meaningful comparison across studies. Most existing datasets focus on a single cultivar or a single controlled storage condition, which restricts their representativeness. Future work should therefore prioritize systematic collection of time-resolved VOC profiles across multiple grain species, geographical origins, harvest years, and storage environments. Heterogeneous datasets of this kind would enable the identification of both universal and species-specific aging markers and provide the foundation for models with improved generalizability. Database construction should also be accompanied by mechanistic validation of key VOC markers. Integrating VOC profiling with complementary approaches, such as metabolic pathway analysis or sensory-linked validation, will help move beyond purely correlation-based marker selection and support clearer causal interpretation between VOC dynamics and quality deterioration.

### 5.2. Model Innovation: New Paths to Enhance Generalization and Robustness

Beyond data quality and scale, improving model generalization remains a central challenge in VOC-based grain aging studies. Heavy reliance on small, homogeneous laboratory datasets often produces overly optimistic performance estimates that do not translate to real-world storage environments. A major contributor to this problem is widespread use of AA protocols. Although AA experiments are essential for generating time-resolved data within feasible timeframes, they may alter the kinetics and relative pathways of VOC formation compared with NA. Under accelerated conditions, VOC accumulation often reflects stress-induced oxidative reactions rather than the gradual metabolic and physicochemical evolution that occurs during long-term storage. As a result, VOC ratios and temporal trends observed in AA studies may not mirror those in commercial storage. Biomarkers that perform well under AA conditions may therefore lose predictive relevance in the field. This domain shift reduces ML model transferability and limits practical application. Future modeling research should therefore emphasize robustness across grain varieties, storage environments, and timeframes, rather than maximizing classification accuracy within narrowly defined datasets. One important direction is the development of models that can learn effectively from limited or imbalanced data while minimizing overfitting. When physical samples are scarce, data augmentation strategies such as Synthetic Minority Over-sampling Technique (SMOTE) or Generative Adversarial Networks (GANs) can expand the diversity of training distributions. To further control model complexity in high-dimensional VOC datasets, regularization techniques such as L1 or L2 penalties and ensemble learning approaches such as RF and XGBoost should be prioritized. These methods often demonstrate greater stability than highly parameterized DL models trained on AA-derived datasets. Knowledge transfer strategies, including transfer learning across related grain species or aging regimes, also offer promise for mitigating distributional mismatch between AA and NA when labeled samples are limited. Equally important is the adoption of rigorous validation frameworks. Robust evaluation should include nested cross-validation, independent external validation sets drawn from different harvest years and geographical origins, and statistical resampling methods such as bootstrapping to evaluate performance stability [116]. These practices are essential to prevent performance overestimation, which is common when only homogeneous laboratory datasets are used. Finally, interpretability is increasingly recognized as a critical for adoption in food safety and regulatory contexts. Explainable Artificial Intelligence (XAI) tools, such as SHapley Additive exPlanations (SHAP) and feature attribution analyses, allow model outputs to be linked back to specific VOCs associated with lipid oxidation, Maillard reactions, or microbial metabolism. This transparency strengthens biomarker traceability, supports regulatory acceptance, and builds trust by allowing practitioners to verify the biochemical relevance of automated alerts. Ultimately, successful deployment will depend on models that remain robust and interpretable under real storage conditions, where multiple confounding factors such as temperature fluctuations, humidity gradients, microbial activity, and insect infestation coexist.

### 5.3. System Integration: Bridging the Chasm from Laboratory to Engineering Application

**Figure 3:**
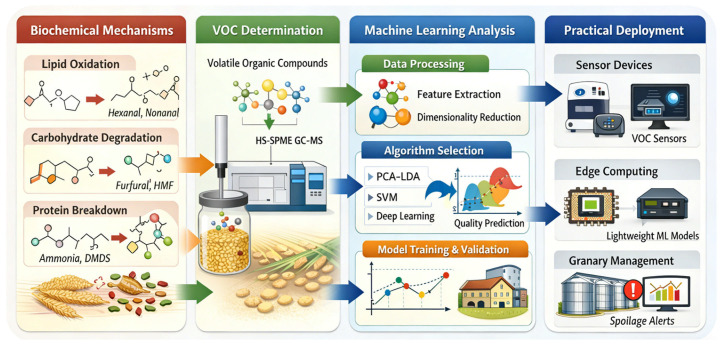
Integrative schematic bridging grain biochemical mechanisms, VOC detection, ML analysis, and industrial deployment.

The crucial step from excellent laboratory performance to reliable, automated deployment in operational granaries depends on end-to-end system integration and rigorous engineering validation. The main challenge is the substantial gap between sophisticated laboratory analysis workflows and the complex; highly variable conditions found in real storage environments. There is an urgent need to develop in situ, rapid, and automated detection systems to replace the current reliance on manual, offline sampling. To illustrate a pathway toward this goal, an integrative schematic is presented in Figure 3. This roadmap explicitly links the biochemical mechanisms described in Figure 1 and the VOC evolution patterns shown in Figure 2 to the downstream ML framework and sensor deployment strategy. In doing so, it ensures that engineering design decisions remain grounded in biological reality. The first requirement is innovation in sensor hardware. Development efforts should focus on miniaturized gas-sensor units that combine high sensitivity with high stability and that operate together with automated headspace sampling systems. Guided by the framework in Figure 3, sensor arrays should be tuned to capture high-impact biomarkers, such as hexanal and nonanal, that dominate the dynamic accumulation patterns shown in Figure 2. The objective is to translate the complex GC-MS laboratory workflow into an embedded device that can be deployed directly within the grain bulk. This represents a shift from bringing the sample to the laboratory toward bringing the sensor to the grain. Such a shift is essential for overcoming the critical limitations caused by non-uniform VOC distribution in silos and the unrepresentative nature of manual sampling. On the algorithmic side, models must be reconstructed in a lightweight form for edge computing. Techniques such as pruning and quantization should be applied to reduce computational and storage demands while maintaining predictive performance. This allows systems to meet the response-time requirements of on-site, real-time analysis and early warning. At the same time, lightweight models must retain key XAI functions so that on-site diagnostic outputs are accompanied by interpretable justifications, facilitating immediate action by granary operators. By tracing automated alerts back to specific metabolic pathways, such as lipid oxidation or microbial activity described in Figure 1, operators can verify the biological relevance of the risk before taking action. System reliability can be further improved by integrating data from auxiliary sensors, including environmental temperature, humidity, and CO_2_ concentration. These additional signals help correct for environmental fluctuations and enhance the robustness of the final diagnostic conclusions. Ultimately, the effectiveness of any integrated system must be demonstrated through rigorous, large-scale engineering validation. This requires long-term stability testing in operational granaries that differ in type, geographic region, and grain variety. System performance should be assessed under real-world disturbances, including diurnal temperature cycles, seasonal changes, and ventilation operations. These conditions cannot be replicated through short-term laboratory testing.

## 6. Conclusions and Outlook

### 6.1. Research Summary

The aging of grains during storage is a complex biochemical process involving multiple pathways, including lipid oxidation, the Maillard reaction, and microbial metabolism. The resulting quality deterioration poses a persistent challenge to global food security and resource efficiency. This review has systematically examined the research framework for the intelligent discrimination of grain aging based on the integration of VOC fingerprinting with ML techniques. Compared with conventional physicochemical indicators, VOCs capture the molecular progression of early-stage aging with higher sensitivity and specificity. They therefore serve as ideal biomarkers for early warning systems. GC-MS remains the cornerstone analytical platform for resolving complex VOC fingerprints because of its high resolution and sensitivity. In parallel, HS-SPME has become the mainstream sample preparation technique due to its efficiency and operational flexibility. Critically, the application of ML, ranging from shallow models (e.g., PLS-DA and SVM) to DL algorithms (e.g., CNNs), has addressed the core challenges of feature extraction, dimensionality reduction, and non-linear modeling inherent in high-dimensional VOC data. This synergy has enabled precise discrimination of aging stages, identification of the root causes of deterioration, and even inference of geographical provenance. Nevertheless, the field remains constrained by several bottlenecks: insufficient methodological standardization, limited sample sizes, weak model generalizability, and a marked lack of validation in complex, real-world environments. Together, these challenges hinder the transition of this technology from laboratory research to large-scale practical application.

### 6.2. Overall Outlook

Despite the remaining challenges, the “VOC fingerprinting-ML” paradigm clearly represents the future direction of grain storage quality monitoring. Its development is driving a fundamental shift in the field, moving away from traditional approaches that rely on manual experience and endpoint assays, and toward a modern framework that is automated, intelligent, and real-time. Looking ahead, progress in this domain will depend on deep, multi-disciplinary integration across multiple technologies. First, the standardization of methodologies and the construction of large-scale, shared databases form the foundation on which all generalizable models must be built. Second, next-generation ML methods, including transfer learning, few-shot learning, XAI, and multi-modal data fusion (e.g., integrating spectral and imaging data), will be critical for improving model robustness and generalizability. Ultimately, the value of this technology must be demonstrated through engineering application. Achieving this will require coordinated innovation in portable detection devices, embedded systems, Internet of Things (IoT) technology, and intelligent algorithms to reach the final goal of real-time, online, non-destructive monitoring of the granary. In summary, the deep integration of VOCs and ML offers a novel research perspective for elucidating the mechanisms of grain aging and provides a solid technical foundation for building reliable, efficient intelligent storage management systems. With continued advances in technological innovation and system integration, this framework is poised to play a vital role in the global food supply chain, particularly in quality assurance, loss reduction, efficiency improvement, and risk management. It represents a powerful scientific and technological pillar to support responses to future food security challenges.

## 7. Literature Search and Selection Strategy

To ensure a comprehensive and reproducible review, a systematic literature search was conducted using major scientific databases, including Web of Science and Google Scholar. The search strategy employed Boolean operators to combine keywords related to the core topics: (“grain” OR “cereal”) AND (“aging” OR “storage deterioration”) AND (“volatile organic compounds” OR “VOCs”) AND (“machine learning” OR “chemometrics”). The selection criteria prioritized peer-reviewed research articles and reviews published primarily within the last 15 years to capture recent technological advancements, while seminal studies establishing fundamental principles were also retained. Articles were evaluated based on their relevance to VOC generation mechanisms, analytical techniques, and data processing algorithms. Non-English publications and studies lacking sufficient methodological detail were excluded to ensure the quality of the review.

## Figures and Tables

**Table 1:** Comparison of different VOC determination techniques.

Method	Detection Principle	Sensitivity	Advantages and Limitations	References
GC-FID	Ion current produced by the combustion of organics in a hydrogen flame	High (ppb level, especially for hydrocarbons)	Low cost, wide linear range, ideal for quantification but lacks structural information	[48,53]
GC-IMS	Chromatographic separation combined with ion mobility spectrometry	ppb-ppt	Rapid response, minimal pretreatment, suitable for fast screening but limited for compound identification, database-dependent	[50,54]
GC-O	Human olfaction synchronized with chromatographic peaks	Depends on human sensitivity	Direct evaluation of odor activity, identifies key aroma compounds, but highly subjective and difficult to standardize	[45,49]
GC-MS	Mass-based molecular structure analysis following chromatographic separation	ppb-ppt	High sensitivity and selectivity, enables identification of unknown compounds, but costly and time-consuming	[51,52,55]

Note: GC-FID, gas chromatography flame ionization detection; GC-IMS, gas chromatography-ion mobility spectrometry; GC-O, gas chromatography-olfactometry; GC-MS, gas chromatography-mass spectrometry.

**Table 2:** Comparison of different ML models applied in VOC-based discrimination.

Model	Algorithm Characteristics	Advantages	Limitations	References
PLS-DA/PCA-LDA	Supervised learning; seeks linear combinations that maximize inter-group variance	High interpretability; suitable for key variable screening	Restricted to linear relationships; may cause information loss and weak generalization with complex, non-linear data	[83,84,85]
SVM	Classifier based on kernel functions	Suitable for small-sample, high-dimensional data; clear class boundaries; good robustness	Sensitive to parameter tuning (e.g., kernel selection); computational complexity limits scalability for large datasets	[86,87]
RF	Ensemble of multiple decision trees	High accuracy; capable of estimating variable importance; robust and less prone to overfitting	Can exhibit bias with highly correlated predictors; ensemble structure is difficult to interpret (black-box nature)	[88,89]
XGBoost	Optimized version of gradient boosting decision tree	High accuracy; effective for sparse data; fast computational efficiency	Demands extensive parameter tuning; sensitive to class imbalance; risk of overfitting noisy datasets	[90,91]
DL	Automatic extraction of nonlinear features	Powerful feature learning and complex pattern recognition capabilities	Requires large datasets and computational resources; limited interpretability; potential training inefficiency with sparse data	[92,93]

Note: PLS-DA, partial least squares discriminant analysis; PCA-LDA, Principal Component Analysis–Linear Discriminant Analysis; SVM, support vector machines; RF, random forest; XGBoost, eXtreme Gradient Boosting; DL: Deep learning.

**Table 3:** Species-Specific VOCs and modeling strategies.

Grain Type	Dominant Aging Mechanism	Key Biomarkers	Recommended Modeling Methods	References
Rice	Aroma degradation and Bran lipid oxidation	2-AP; Hexanal, Pentanal, Decanal	SVM, RF	[59,60,104]
Maize	Rapid oxidation of germ lipids	Nonanal, Hexanal, 2-Heptanone	PLS-DA, PCA-LDA	[101,102,103,104]
Wheat	Mild oxidation and Enzymatic activity	(E)-2-Nonenal, 1-Octanol, Hexanal	DL (for large datasets), RF	[104,107]

Note: All GC–MS-derived VOC datasets require appropriate preprocessing, including peak deconvolution, retention-time alignment, normalization, and model validation.

## Data Availability

No new data were created or analyzed in this study.
